# Physiological roles of sigma factor SigD in *Corynebacterium glutamicum*

**DOI:** 10.1186/s12866-017-1067-6

**Published:** 2017-07-12

**Authors:** Hironori Taniguchi, Tobias Busche, Thomas Patschkowski, Karsten Niehaus, Miroslav Pátek, Jörn Kalinowski, Volker F. Wendisch

**Affiliations:** 1 Genetics of Prokaryotes, Faculty of Biology, Bielefeld University, Bielefeld, Germany; 2 Center for Biotechnology, Bielefeld University, Bielefeld, Germany; 3 Proteome and Metabolome Research, Faculty of Biology, Bielefeld University, Bielefeld, Germany; 4 Institute of Microbiology, Academy of Sciences of the Czech Republic, Prague, Czech Republic

**Keywords:** *Corynebacterium glutamicum*, Sigma factor, SigD, Mycomembrane, Trehalose dicorynomycolate

## Abstract

**Background:**

Sigma factors are one of the components of RNA polymerase holoenzymes, and an essential factor of transcription initiation in bacteria. *Corynebacterium glutamicum* possesses seven genes coding for sigma factors, most of which have been studied to some detail; however, the role of SigD in transcriptional regulation in *C. glutamicum* has been mostly unknown.

**Results:**

In this work, pleiotropic effects of *sigD* overexpression at the level of phenotype, transcripts, proteins and metabolites were investigated. Overexpression of *sigD* decreased the growth rate of *C. glutamicum* cultures, and induced several physiological effects such as reduced culture foaming, turbid supernatant and cell aggregation. Upon overexpression of *sigD*, the level of Cmt1 (corynomycolyl transferase) in the supernatant was notably enhanced, and carbohydrate-containing compounds were excreted to the supernatant. The real-time PCR analysis revealed that *sigD* overexpression increased the expression of genes related to corynomycolic acid synthesis (*fadD2*, *pks*), genes encoding corynomycolyl transferases (*cop1*, *cmt1*, *cmt2*, *cmt3*), L, D-transpeptidase (*lppS*), a subunit of the major cell wall channel (*porH*), and the envelope lipid regulation factor (*elrF*). Furthermore, overexpression of *sigD* resulted in trehalose dicorynomycolate accumulation in the cell envelope.

**Conclusions:**

This study demonstrated that SigD regulates the synthesis of corynomycolate and related compounds, and expanded the knowledge of regulatory functions of sigma factors in *C. glutamicum*.

**Electronic supplementary material:**

The online version of this article (doi:10.1186/s12866-017-1067-6) contains supplementary material, which is available to authorized users.

## Background

Sigma factors are a component of bacterial RNA polymerase holoenzymes essential for promoter recognition and transcription initiation [1]. Most bacteria encode multiple sigma factors, and each sigma factor containing RNA polymerase holoenzyme initiates transcription from the cognate promoter sequences [2–5]. By replacing a sigma factor in RNA polymerase holoenzyme, bacteria activate transcription of a different gene set under different conditions, and cope with environmental changes [6]. Therefore, sigma factors play an important role in transcriptional regulation in a global manner. The knowledge of regulations by sigma factors is helpful to elucidate the regulatory network of the organism.

*Corynebacterium glutamicum* was first isolated as an organism secreting high amounts of L-glutamate [7]. Nowadays, this bacterium is used for production of L-amino acids in million tons per year, especially L-glutamate and L-lysine [8]. *C. glutamicum* ATCC 13032 has seven sigma factor genes in its chromosome, *sigA*, *sigB*, *sigC*, *sigD*, *sigE*, *sigH* and *sigM* [9, 10]. The physiological functions of SigA, SigB, SigC, SigE, SigH and SigM have been studied to some extent [11]; however, the regulation and physiological roles of SigD in *C. glutamicum* have not yet been revealed. *sigD* gene is well conserved among corynebacteria, and 17 out of 19 examined *Corynebacterium* species possess *sigD* genes [11]. Therefore, it is assumed that SigD plays a substantial role in transcriptional regulation and subsequent adaptation of *C. glutamicum* to changing environments.

*C. glutamicum* belongs to the CMN (*C* *orynebacterium*, *M* *ycobacterium*, *N* *ocardia*) group, which is characterized by the unique molecular constituents of their cell envelopes such as the mycomembrane [12]. The mycomembrane is composed of a monolayer of corynomycolate (α-alkyl, β-hydroxy fatty acid), which is covalently linked to arabinogalactan or forms other lipids such as trehalose monocorynomycolate (TMCM) and trehalose dicorynomycolate (TDCM) [13].

In this work, we evaluated the effects of deletion and overexpression of *sigD* on the cell phenotype, and revealed the influence of *sigD* overexpression on transcripts, proteins and metabolites. The achieved results disclosed the important roles of SigD in mycomembrane synthesis and maintaining cell wall integrity.

## Methods

### Bacterial strains, plasmids and oligonucleotides

The strains, plasmids and oligonucleotides used in this work are listed in Additional file 1: Table S1. A plasmid for *sigD* overexpression was constructed based on pVWEx1, which is an IPTG inducible expression vector for *E. coli* and *C. glutamicum* [14]. A plasmid for gene disruption was constructed based on pK18mobsacB [15]*.* For plasmid construction, DNA fragments were amplified from the genomic DNA of *C. glutamicum* ATCC 13032 by PCR with the oligonucleotide pairs shown in Additional file 1: Table S1. These fragments were inserted into the digested plasmid by ligation or Gibson assembly [16, 17]. *E. coli* DH5α was used for cloning. *E. coli* competent cells were transformed by the heat shock method [16] or by the electroporation method [18]. DNA sequences of all cloned DNA fragments were confirmed to be correct by sequencing. *C. glutamicum* competent cells were transformed by electroporation at 2.5 kV, 200 Ω, and 25 μF [8, 19]. Gene disruption via two-step homologous recombination and the following selections were carried out as previously described [8]. Disruption was verified by PCR with the respective oligonucleotide pairs.

### Medium and conditions for growth experiments

Unless otherwise specified, *C. glutamicum* cells were precultured overnight in lysogeny broth (LB) medium [16] supplemented with 56 mM of glucose, washed once with chemically defined CGXII medium [8] without carbon source, and then inoculated into CGXII medium with 222 mM of glucose at an initial OD_600_ of 1. The cultivation was performed at 30 °C, 120 rpm. OD_600_ was measured with UV-1202 spectrophotometer (Shimadzu, Duisburg, Germany) with suitable dilutions. When necessary, 25 μg/mL of kanamycin and appropriate concentrations of IPTG were added as indicated in the text. For the growth experiment in BioLector® cultivation system (m2pLabs, Baesweiler, Germany), cells were cultivated in 1 mL of CGXII medium with 222 mM of glucose using FlowerPlate® (m2pLabs, Baesweiler, Germany) at 30 °C, 1100 rpm. Cell growth was monitored online every 10 min, and the maximum growth rate (h^−1^) was determined from the growth rates μ (h^−1^) which were calculated with regression analysis from backscattering light intensity (wavelength of 620 nm) at 20 consecutive measuring points.

### Photometric determination of supernatant turbidity and observation of cell aggregation by microscopy

To quantify turbidity of supernatants, cell cultures were centrifuged for 30 min with 15,000 x *g* at room temperature. The absorption of supernatants was measured at a wavelength of 600 nm. For microscopic imaging, the culture of each strain in the stationary phase was diluted in CGXII medium without carbon source to an OD_600_ of 1, and observed by microscopy with 100 x oil immersion objective lens and 10 x ocular lens. Quantification of cell aggregate size was performed using ImageJ (https://imagej.nih.gov/ij/).

### Protein analysis of the supernatant

Supernatants were taken from the stationary phase cultures. Four volumes of acetone were added to one volume of supernatant, and stored at −20 °C overnight. After centrifugation at 4 °C, 20,000 x *g* for 15 min, precipitates were resuspended in 20 mM Tris-HCl (pH 7). SDS-PAGE was performed using Tris-glycine discontinuous buffer, and visualized by staining with Coomassie Brilliant Blue R250 as previously described [16]. Quantification of the intensity of each band was performed using ImageJ. Protein bands with different intensities observed in the control strain and the *sigD* overexpressing strain were excised from SDS-PAGE gels and transferred to a new tube which had been washed with trifluoroacetic acid: acetonitrile: H_2_O (0.1:60:40 *v*/v) in advance. The digestion of protein in the excised band was performed with trypsin overnight as previously described [20]. Protein sequences were identified using an ultrafleXtreme MALDI-TOF/TOF mass spectrometry (Bruker, Bremen, Germany) and Mascot search engine (Matrix Science, London, UK) as previously described with some modifications for *C. glutamicum* ATCC 13032 [21].

### Quantification of carbohydrate in acetone precipitates

Supernatants were precipitated with acetone as described above. Precipitates were resuspended with 100 μL of 20 mM Tris-HCl (pH 7), placed at room temperature for 30 min, and then the insoluble fraction was recovered by centrifugation. Insoluble fractions were hydrolyzed as previously described [22]. Briefly, the precipitates were resuspended with 75 μL of 72% (*w*/w) sulfuric acid and incubated at room temperature for 3 h. The slurry was diluted to 1 mL with water, heated at 100 °C for 4 h, and cooled down on ice. Colorimetric quantification for carbohydrates was performed by the phenol sulfuric acid method as previously described with some modifications [23, 24]. Briefly, 200 μL of hydrolysate was mixed with 600 μL of concentrated sulfuric acid rapidly, and 120 μL of 5% phenol (*w*/*v*) in water was added immediately. The mixture was incubated for 5 min at 90 °C, and cooled to room temperature for 5 min. The absorption at 490 nm was measured, and compared to the absorption of the control samples with different concentrations of arabinose.

### RNA extraction

Cells were first precultured in LB medium, and inoculated in CGXII medium with 222 mM of glucose for adaptation. Then, the appropriate amount of cell culture was inoculated into fresh CGXII medium with 222 mM of glucose at an initial OD_600_ of 1. The cells were harvested at an OD_600_ between 6 and 8. Cell cultures (1 mL) were centrifuged for 30 s at 20,000 x *g*, and immediately frozen with liquid nitrogen after removing supernatant. RNA isolation was performed using the RNeasy mini kit along with the RNase-free DNase set (Qiagen, Hilden, Germany) as previously described [25]. The absence of contaminating genomic DNA in RNA samples was confirmed by PCR with multiple oligonucleotide pairs specific to genomic DNA.

### RNA isolation, library preparation and RNA-seq

For transcriptome sequencing, RNA samples isolated individually from biological triplicates were mixed for each stain. Quality check of the isolated RNA, library preparation, RNA-seq and data analysis were performed as previously described [26]. Briefly, RNA quality was checked by Trinean Xpose (Gentbrugge, Belgium) and Agilent RNA Nano 6000 kit with Agilent 2100 Bioanalyzer (Agilent Technologies, Böblingen, Germany). Ribo-Zero rRNA Removal Kit (Bacteria) from Illumina (San Diego, CA, USA) was used to remove the ribosomal RNA molecules from the isolated total RNA. Removal of rRNA was checked by Agilent RNA Pico 6000 kit on Agilent 2100 Bioanalyzer (Agilent Technologies, Böblingen, Germany). TruSeq Stranded mRNA Library Prep Kit from Illumina (San Diego, CA, USA) was used to prepare cDNA libraries. cDNAs were sequenced paired end on an Illumina MiSeq system (San Diego, CA, USA) using 50 bp read length.

### Read mapping, data visualization and analysis of gene expression

Trimmed reads (26 nt) were mapped to the *C. glutamicum* ATCC 13032 reference genome sequence [9] with SARUMAN [27], allowing for up to one error per read. The forward and reverse read, if both present and with a maximum distance of 1 kb, were combined to one read that contains the reference sequence as insert. Paired mappings with a distance >1 kb were discarded, and paired reads with either only the forward or only the reverse read mapping were retained as single mapping reads, as previously described [28]. ReadXplorer 2.2.0 was used for visualization of short read alignments [29]. For differential gene expression analysis, ReadXplorer and Bioconductor package DESeq implemented in ReadXplorer were used [29, 30]. Genes with the mean value of signal intensity less than 30 were discarded. A-value and M-value of each gene were calculated based on the intensity value of the strain of interest and the control strain.

### Real-time PCR analysis

For real-time PCR analysis, relative abundance of mRNA of each gene was quantified using the same amount of total RNA. RNA sample extracted from biological triplicates were quantified individually. The experiment and analysis were performed as previously described with respective oligonucleotide pairs in Additional file 1: Table S1 [31].

### Detection of trehalose dicorynomycolate by thin layer chromatography

Lipid extraction and thin layer chromatography (TLC) were performed based on the method previously described [32, 33]. Briefly, the crude lipid was extracted from cell pellet with CHCl_3_/CH_3_OH (1:1 *v*/v) once, and with CHCl_3_/CH_3_OH (2:1 *v*/v) for three times. All the extracts were pooled for each strain, and mixed with CHCl_3_/CH_3_OH/H_2_O (8:4:2 *v*/v) resulting the aqueous layer and the organic layer. The lower organic layer was collected and evaporated to dryness. The dried lipid was weighed and resuspended with CHCl_3_/CH_3_OH (4:1 *v*/v) to the same concentration for each sample. TLC was performed with ALUGRAM SIL G/UV254 (Macherey-Nagel, Germany) with CHCl_3_/CH_3_OH/H_2_O (30:8:1 *v*/v) as a developing solvent. Total lipid (600 μg) was developed for each strain, and the bands were visualized by spraying TLC plate with sulfuric acid and heating to 110 °C. The mobility of TDCM or TMCM was determined by the retardation factor (Rf) value from the previous studies [32, 33]. Quantification of the intensity of each band was performed with ImageJ.

## Results

### Deletion and overexpression of *sigD* influenced the maximum growth rate

**Fig. 1:**
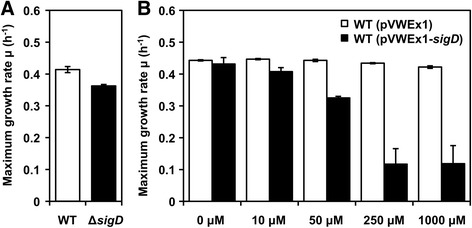
Maximum growth rates of the Δ*sigD* strain and the *sigD* overexpressing strain with different IPTG concentrations. The maximum specific growth rate (h^−1^) was shown for (**a**) *C. glutamicum* WT and Δ*sigD,* and (**b**) WT(pVWEx1) and WT(pVWEx1-*sigD*) with different IPTG concentrations (0, 10, 50, 250, 1000 μM). Error bars represent standard deviations from biological triplicates

The *sigD* deletion mutant (Δ*sigD*) and the *sigD* overexpressing strain were cultivated in CGXII medium containing 222 mM of glucose as carbon source. *sigD* was overexpressed from the plasmid pVWEx1-*sigD* using an IPTG inducible promoter and different IPTG concentrations (0, 10, 50, 250 or 1000 μM). The Δ*sigD* mutant grew slightly slower than the wild type (WT) strain (Fig. 1a). The maximum growth rate of the *sigD* overexpressing strain WT(pVWEx1-*sigD*) decreased in an IPTG-dependent manner, which was not observed for WT(pVWEx1) (Fig. 1b). Higher concentrations of IPTG (250 and 1000 μM) severely inhibited the growth of WT(pVWEx1-*sigD*). The final biomass concentrations after 24 h of cultivation were comparable for WT, Δ*sigD*, WT(pVWEx1) and WT(pVWEx1-*sigD*) with 0, 10 or 50 μM of IPTG (data not shown). The slower growth of the Δ*sigD* strain indicated that *sigD* is beneficial for growth in minimal CGXII medium although it is not essential for growth under optimum conditions. As excessive *sigD* overexpression at the high IPTG concentrations was found to be harmful to cells, induction with 50 μM of IPTG was used for further experiments.

### *sigD* overexpression influenced cell culture characteristics

**Fig. 2:**
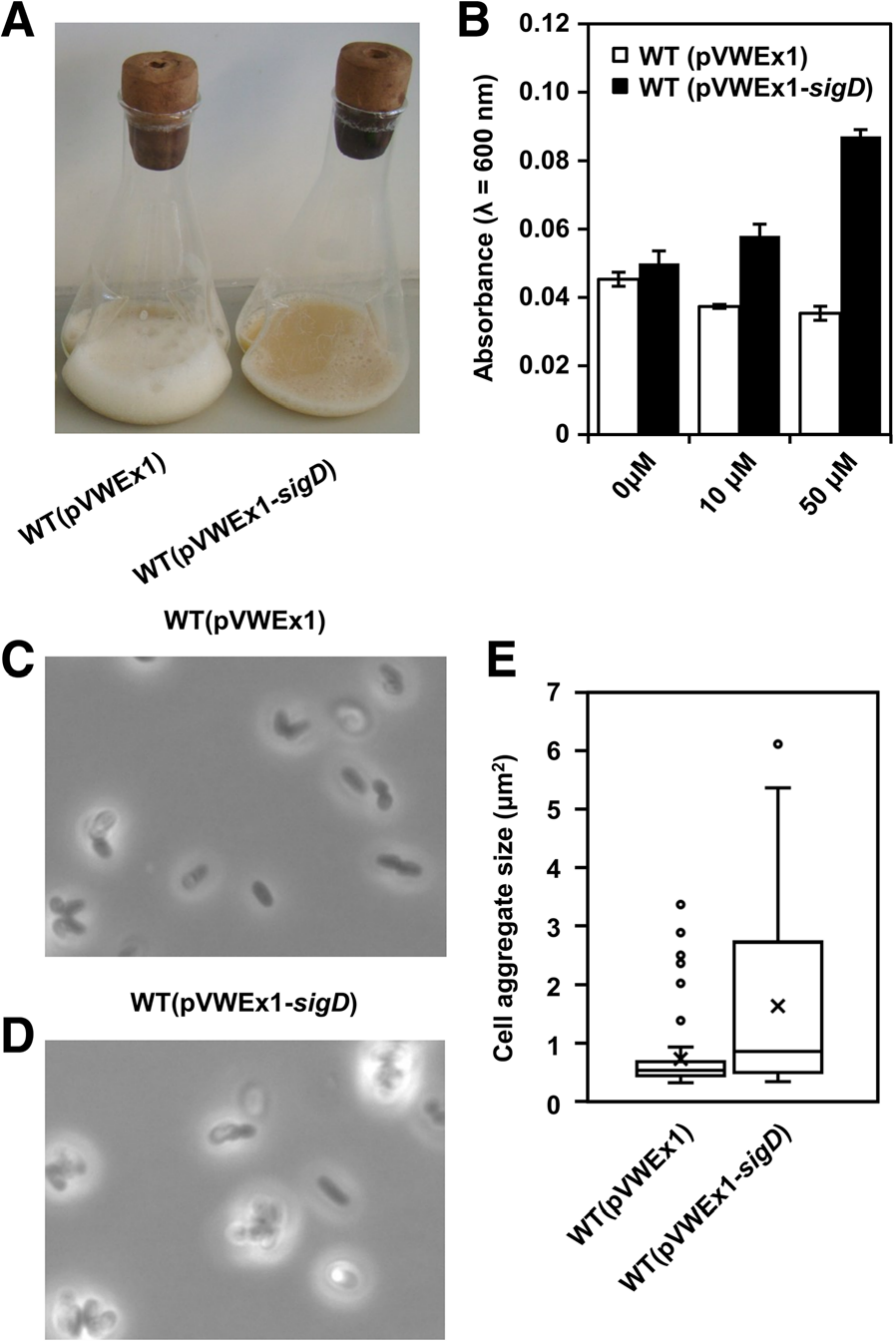
Influence of *sigD* overexpression on cell cultures and cell morphology. **a** Cell cultures with 50 μM of IPTG after 36 h of cultivation are shown. **b** Supernatant turbidity with different concentrations of IPTG (0 μM, 10 μM, 50 μM) after 36 h is shown. Error bars represent standard deviations from biological triplicates. Microscopic images of the WT strain (**c**) and the *sigD* overexpressing strain (**d**) are shown. Cells in the stationary phase were observed under the microscope with a magnification of 1000. **e** Distribution of the size of cell aggregates is shown. The size of cell aggregates was analyzed by ImageJ, and the distribution was visualized by the box-and-whisker plot. Lower whisker, lower quantile, median, upper quantile and upper whisker are shown. The cross point indicates mean, and outliners were plotted as individual points

WT(pVWEx1-*sigD*) with 50 μM of IPTG showed distinct characteristics compared to WT(pVWEx1) as the cultures of WT(pVWEx1-*sigD*) foamed significantly less than those of WT(pVWEx1) (Fig. 2a). Cell cultures with different IPTG concentrations (0, 10, 50 μM) using FlowerPlates and a BioLector system revealed that the supernatants of WT(pVWEx1-*sigD*) cultures showed higher turbidity than those of the control strain, and this turbidity increased in an IPTG-dependent manner (Fig. 2b). In addition to these distinct characteristics of the cell culture, cell aggregation was observed under the microscope in WT(pVWEx1-*sigD*) with IPTG (Fig. 2c–e). Taken together, overexpression of *sigD* induced pleiotropic changes of the phenotype of *C. glutamicum*.

### *sigD* overexpression changed the pattern of secreted proteins

**Fig. 3:**
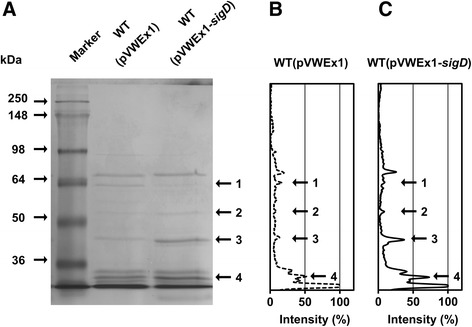
1D-SDS PAGE of proteins in the supernatants. **a** Secreted proteins were analyzed by 12% SDS-PAGE. The molecular sizes of proteins in the marker are shown in kDa. WT(pVWEx1) and WT(pVWEx1-*sigD*) protein samples were obtained by acetone precipitation of the supernatants. Proteins from 200 μL of the supernatant was loaded on each lane. The intensity of secreted protein bands was quantified for WT(pVWEx1) (**b**) and for WT(pVWEx1-sigD) (**c**). The highest intensity of the band was normalized to 100%. The protein bands labeled with numbers were subjected to MALDI-TOF/TOF MS. 1: Psp3 (Cg2061), 2: LppS (Cg2720), 3: Cmt1 (Cg0413), 4: Cmt2 (Cg3186) and Cg2052

Overexpression of *sigD* influenced the culture characteristic of foaming. Therefore, the profile of proteins in the culture supernatant was compared between WT(pVWEx1) and WT(pVWEx1-*sigD*). Proteins in the supernatants were analyzed by 1D SDS-PAGE after concentrating by acetone precipitation. We observed different band patterns of proteins secreted by WT(pVWEx1) and WT(pVWEx1-*sigD*) (Fig. 3a). The proteins in the bands with different intensity were further characterized by tryptic digestion and MALDI-TOF/TOF mass spectrometry (Fig. 3b, c). In this way, corynomycolyl transferase Cmt1 was identified in the band 3, which showed a higher intensity when *sigD* was overexpressed. The band 2 was identified as L, D-transpeptidase LppS, and the band 4 was identified as mixture of two proteins, corynomycolyl transferase Cmt2 and putative secreted protein Cg2052. On the other hand, Psp3, which was found in the band 1 and annotated as putative secreted protein, was less abundant in WT(pVWEx1-*sigD*). These results demonstrated that overexpression of *sigD* altered the secreted protein profile. Since Cmt1 abundance in the supernatant increased most upon *sigD* overexpression (Fig. 3a, c), *cmt1* was overexpressed in the WT strain. However, less foaming was not observed under these conditions (data not shown). Therefore, we concluded that less foaming of the culture did not occur only due to Cmt1 protein abundance but to complex or other unknown reasons.

### *sigD* overexpression induced the secretion of carbohydrate-containing compounds

During acetone precipitation of the supernatant, we observed an insoluble fraction only in the sample of the *sigD* overexpressing strain. This insoluble fraction still existed after an overnight protease K treatment. For the strain *C. glutamicum* CGL2005, it was reported that the ethanol-precipitated fraction of extracellular components consisted primarily of carbohydrates [34]. Therefore, the carbohydrate content in this fraction was determined by the phenol sulfuric acid method [23], which detects all classes of carbohydrates [35]. Arabinose, which is one of the components of peptidoglycan in *C. glutamicum,* was used as control. Colorimetric determination after hydrolysis confirmed that the insoluble fraction of WT(pVWEx1-*sigD*) supernatants contained carbohydrates (arabinose equivalent of 1.3 mM). On the other hand, the same treatment of WT, Δ*sigD* or WT(pVWEx1) supernatants resulted in carbohydrate contents below the detection limit (arabinose equivalent <0.1 mM) (Table 1). These results showed that *sigD* overexpression induced the secretion of polysaccharides or carbohydrate-containing compounds into the supernatant.

**Table 1:** Carbohydrate content in acetone precipitated culture supernatants

	WT	Δ*sigD*	WT(pVWEx1)	WT(pVWEx1-*sigD*)
Arabinose equivalent carbohydrate (mM)	<0.1	<0.1	<0.1	1.29 ± 0.28

Carbohydrate content was measured by the phenol sulfuric acid method. Arabinose samples with known concentrations were used as standards. Carbohydrate content was calculated to the concentration (mM) of arabinose equivalent carbohydrate. The detection limit is 0.1 mM. Standard deviations were calculated from three biological replicates

### SigD regulated transcription of several genes related to cell envelope integrity

**Fig. 4:**
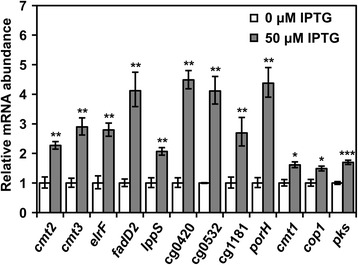
Relative mRNA abundance of genes upregulated during *sigD* overexpression. Relative abundance of mRNA of each gene was quantified by real-time PCR. The white and gray columns show the abundance in WT(pVWEx1-*sigD*) without IPTG (0 μM IPTG), and in WT(pVWEx1-*sigD*) with 50 μM of IPTG (50 μM IPTG), respectively. Error bars represent standard deviations calculated from biological triplicates. The *p*-value of mRNA abundance was calculated by Student’s t-test (two-tail, unpaired) between 0 μM and 50 μM of IPTG, and is shown by *, ** and *** for <0.05, <0.01 and <0.001, respectively

RNA-seq and real-time PCR analysis were performed to understand the effects caused by *sigD* overexpression or deletion at the transcriptional level. The relative abundance of mRNA of each gene was compared in WT(pVWEx1-*sigD*) without IPTG or with 50 μM of IPTG to analyze the effects of *sigD* overexpression. On the other hand, the mRNA abundance was compared between the Δ*sigD* and the WT strain to study the effects of *sigD* deletion. First, RNA - seq analysis was applied to screen the genes whose expression levels changed upon *sigD* overexpression or deletion. RNA-seq analysis implied that expression of 29 genes increased upon *sigD* overexpression (M-value >1) (Additional file 2: Table S2). Of these 29 genes, several genes are annotated as genes related to cell wall integrity; 6 genes (*cop1*, *cmt1*, *cmt2*, *cmt3*, *elrF* and *fadD2*) as corynomycolyl related proteins, a gene annotated as L, D-transpeptidase (*lppS*) and three genes (cg0420, cg0532, cg1181) as glycosyltransferases. In addition, expression of two further genes encoding proteins with mycomembrane-related functions, *porH* (cg3009) and *pks* (cg3178), increased upon *sigD* overexpression (M-value >log_2_(1.5)) and slightly decreased in the Δ*sigD* strain (M-value <−log_2_(1.5)). Based on these RNA-seq results, the expression of those genes was further analyzed by real-time PCR (Fig. 4). All of the tested genes were shown to be upregulated significantly as consequence of *sigD* overexpression. Increased transcript levels of *cmt1*, *cmt2* and *lppS* were consistent with the increased protein levels of Cmt1, Cmt2 and LppS in culture supernatants upon *sigD* overexpression (Fig. 3b, c). These results showed that the function of SigD is related to the regulation of cell envelope integrity such as mycomembrane synthesis and cell wall synthesis.

### Overexpression of *sigD* increased the amounts of trehalose dicorynomycolate

**Fig. 5:**
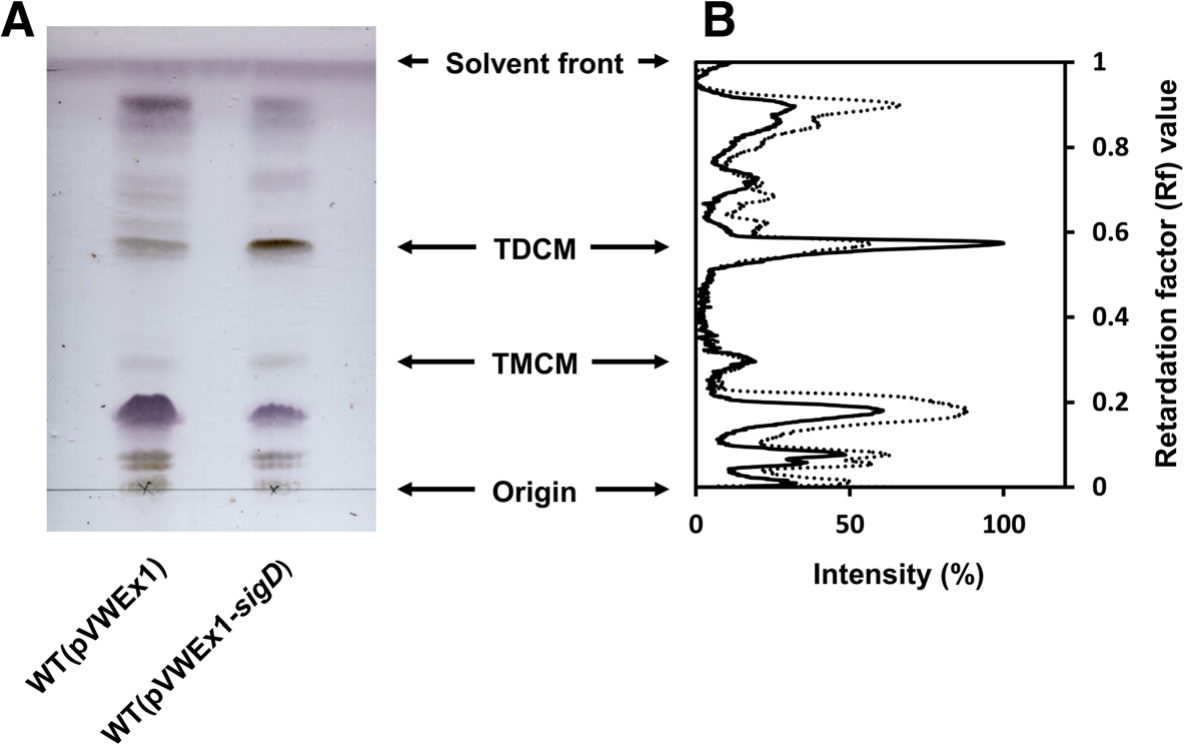
TLC analysis of lipid crude extracts**. a** Lipid crude extracts were analyzed by thin layer chromatography. **b** The intensity of bands was quantified for WT(pVWEx1) and WT(pVWEx1-*sigD*). The highest intensity of the band was normalized to 100%. The dotted and black lines indicate the intensity profile for WT(pVWEx1) and WT(pVWEx1-*sigD*), respectively. The mobility of TDCM (trehalose dicorynomycolate) and TMCM (trehalose monocorynomycolate) were determined by their Rf values taken from the previous studies [32, 33]. CHCl_3_/CH_3_OH/H_2_O (30:8:1 *v*/v) was used as development solvent

The results of the real-time PCR analysis confirmed that the expression of several genes annotated as corynomycolyl transferase (*cop1*, *cmt1*, *cmt2*, *cmt3*) and genes related to corynomycolic acid production (*pks* and *fadD2*) increased due to *sigD* overexpression. Overexpression of *cop1* is known to increase the trehalose dicorynomycolate (TDCM) content in the cells [33]. To confirm the effect of *sigD* overexpression on the TDCM content, the crude lipid was extracted from cells, and its composition was analyzed by TLC (Fig. 5). The intensity of the band corresponding to TDCM increased 39% upon *sigD* overexpression. On the contrary, the intensity of the band corresponding to trehalose monocorynomycolate (TMCM) did not show a notable difference between the two strains. This result indicates that upregulation of multiple genes by *sigD* overexpression increased the flux toward corynomycolic acid synthesis which resulted in an alteration of TMCM/TDCM ratio as well as accumulation of other carbohydrate containing compounds.

## Discussion

**Fig. 6:**
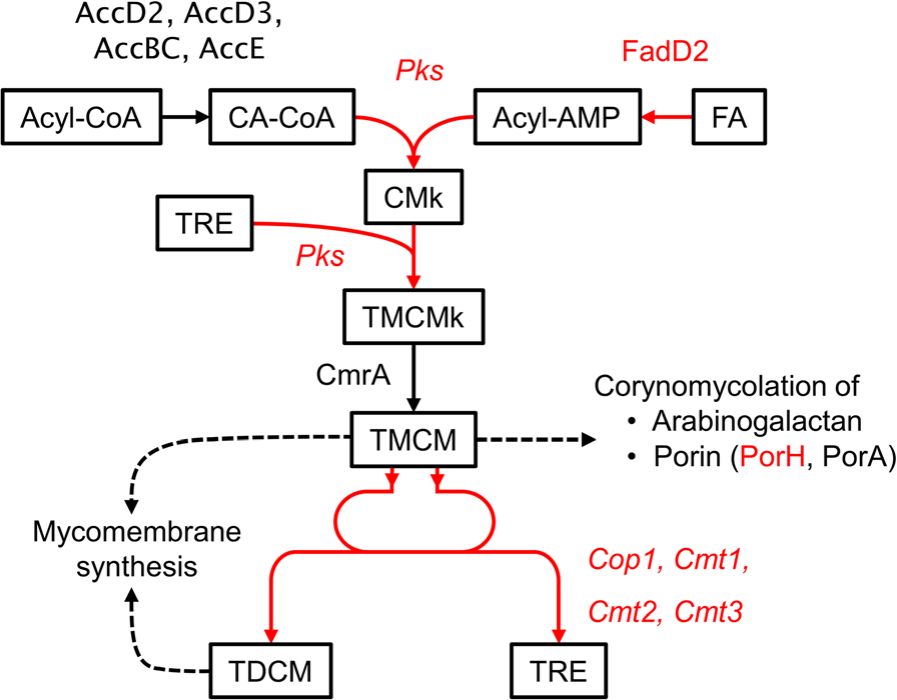
Summary of effects induced by *sigD* overexpression in *C. glutamicum.* Biosynthesis pathway of trehalose dicorynomycolate (*TDCM*) and trehalose monocorynomycolate (*TMCM*) starting from acyl-CoA (*A-CoA*) and fatty acid (*FA*) is shown. The names of enzymes are shown in red letters and the catalyzing reactions are shown in red lines, only when the expression of corresponding genes were confirmed to be upregulated under *sigD* overexpression by transcriptome analysis. *CA-CoA*: carboxylated acyl-CoA, *TRE*: trehalose, *CMk*: Keto corynomycolic acid, *TMCMk*: TMCM keto form

Understanding the regulatory mechanisms of bacteria is important in many fields varying from biotechnology to public health. *C. glutamicum* has been used for the production of amino acids for several decades, however, the transcriptional regulation by sigma factors has not been fully elucidated. As for SigD, selection of high oxygen requiring mutants in a transposon library accidentally revealed that deletion of *sigD* in *C. glutamicum* loses the ability to grow under low oxygen concentrations [36], however, the knowledge is still limited. In the previous works, we demonstrated that overexpression of one of global regulators can artificially perturb the cellular regulation and influence metabolites as well as transcripts [37, 38]. This approach of sigma factor gene overexpression was found to be a useful approach for investigation of regulatory mechanisms and activation of specific biosynthesis pathways in *C. glutamicum*. In this work, deletion and overexpression of *sigD* revealed that sigma factor SigD plays a role in regulating maintenance of the cell wall integrity in *C. glutamicum* (Fig. 6).

The real time PCR analysis revealed that overexpression of *sigD* induced the expression of multiple genes related to mycomembrane synthesis (*cop1*, *cmt1*, *cmt2*, *cmt3*, *elrF*, *fadD2*, *porH* and *pks*) which exist at the different loci in the *C. glutamicum* genome. For mycomembrane biosynthesis, one molecule of fatty acid is carboxylated via the carboxylation complex composed of AccD2, AccD3, AccBC and AccE [39], and a second fatty acid is activated to a fatty acyl-CoA by FadD2 [40]. These two molecules are condensed and attached to trehalose by Pks [41, 42]. This product is reduced to TMCM (trehalose monocorynomycolate) by CmrA [43], and TMCM is exported from cytoplasm [44, 45]. Then, corynomycolyl transferases transfer the corynomycolate group of TMCM onto arabinogalactan, TMCM itself or proteins such as PorH [32, 33, 46, 47]. *C. glutamicum* possesses six corynomycolyl transferase genes, *cop1*, *cmt1*, *cmt2*, *cmt3*, *cmt4* and *cmt5* [32], and Cop1, Cmt1 and Cmt2 catalyze TDCM synthesis from TMCM [32, 46]. Cop1 is also reported to transfer corynomycolate from TMCM to arabinogalactan in the cell wall in *C. glutamicum* CGL2005 [33]. Interestingly, *sigD* overexpression induced the expression of four out of six corynomycolyl transferase genes at the same time, and enhanced the secretion of Cmt1 and Cmt2 to the supernatant. PorH forms the major cell wall channel penetrating the mycomembrane together with the other protein PorA [48], and corynomycolation of PorH and PorA catalyzed by Cmt1 was shown to be necessary for the pore forming activity [47, 49]. Furthermore, ElrF was identified as the envelope lipids regulation factor which regulates lipid composition of corynomycolic acids and phospholipids in cell envelope [50]. In this study, increased content of TDCM in the crude lipid extract indicated that *sigD* overexpression influences not only the transcription of those genes but also the metabolic flux toward mycomembrane synthesis (Fig. 6). These results indicate that SigD controls the integrity of the cell envelope, especially of the mycomembrane in *C. glutamicum*.

SigD of *C. glutamicum* is classified as ECF40 type sigma factor by the ECFfinder program [3], as is SigD of *M. tuberculosis*. SigD in *M. tuberculosis* was shown to be essential for virulence, and inactivation of *sigD* decreased expression of some mycolyl transferase genes as well as other genes related to lipid metabolism and cell wall processes [51, 52]. For example, Calamita et al. reported that the expression of *fbpA* encoding antigen 85A, which is a homolog for corynomycolyl transferase, decreased in the *sigD* deletion strain [52]. Raman et al. showed that the expression of *fbpC* encoding antigen 85C, which is also a homolog for corynomycolyl transferase, decreased two-fold in the *sigD* deletion strain [51]. Even though a different organism has a different regulatory architecture, *C. glutamicum* and *M. tuberculosis* may share the similar regulatory network by SigD.

SigD gene overexpression in *C. glutamicum* led to excretion of carbohydrate-containing compounds and cell aggregation. In *C. glutamicum* CGL2005, various types of polysaccharides were detected extracellularly [34, 53]. In addition, *C. glutamicum* CCTCC M201005 was found in soil as a producer of a bioflocculant consisting of galacturonic acid as the main structural unit [54]. In *M. tuberculosis* and *M. smegmatis*, polysaccharides containing arabinose are suggested to be involved in the aggregation of cells via interaction with Antigen 85s, which are homologs for corynomycolyl transferases [55]. Furthermore, arabinose was shown to promote the cell aggregation of *M. smegmatis* [56]. Therefore, excretion of polysaccharides or carbohydrate-containing compounds caused by *sigD* overexpression may induce cell aggregation also in *C. glutamicum*.

Sigma factors regulate transcription in a global manner and their effects are pleiotropic. In this study, the link between *sigD* overexpression and the regulation of cell wall integrity was confirmed, however, other effects remain to be elucidated, if exist. Furthermore, overexpression of *sigD* turns on the cascade regulation which includes direct and indirect outcomes inside the cells. Based on our results, the physiological role of *C. glutamicum* SigD in cell wall integrity seems apparent, however, the elucidation of the SigD regulon control and definition of the class of SigD-dependent promoters will require further molecular studies.

The non-pathogenic *C. glutamicum* serves as a good model organism for understanding the cell wall biosynthesis and resistance to antibiotics in Corynebacterineae, which include human pathogenic bacteria such as *M. tuberculosis* and *C. diphtheriae* [42]. Therefore, the findings described in this work can be helpful to understand the cell wall biosynthesis of Corynebacterineae. Furthermore, TDCM was shown to induce priming and activation of macrophages in vivo and in vitro in a similar manner as TDM from *M. tuberculosis* [57]. Chemical synthesis of TDM is not easy because it requires multiple steps, and those compounds are extracted from organisms. Considering the common biotechnological use of *C. glutamicum*, the production of TDCM with *C. glutamicum* seems to be an attractive idea. On the other hand, a corynomycolate-less strain is known to excrete more L-glutamate and L-lysine [58] as well as to take up glycerol and acetate more efficiently [33]. Reorganization of the mycomembrane by controlling *sigD* expression can be therefore helpful to understand the permeability barrier of *C. glutamicum* cells, and construct strains with higher or lower permeability barriers.

## Conclusion

In this work, the functions of *C. glutamicum* sigma factor SigD were studied by overexpression or disruption of *sigD* gene. Overexpression of *sigD* led to the several physiological changes such as slower growth, cell aggregation, less foaming of the culture and increased turbidity of the supernatant. The real-time PCR analysis confirmed that overexpression of *sigD* induced the expression of several genes related to maintenance of cell envelop integrity and mycomembrane biosynthesis. Furthermore, overexpression of *sigD* increased the content of trehalose dicorynomycolate in the lipid extract.

## Additional files

Additional file 1: **Table S1.** Bacterial strains, plasmids and oligonucleotides used in this work. Bacterial strains, plasmids and oligonucleotides used in this work are listed. (DOCX 17 kb)

Additional file 2: **Table S2.** RNA-seq analysis of genes differentially transcribed upon sigD overexpression or sigD disruption. The name of genes which expression levels increased under *sigD* overexpression (M-value > 1.0) are listed. (DOCX 18 kb)

## Abbreviations

IPTG: Isopropyl β-D-1-thiogalactopyranoside
TDCM: Trehalose dicorynomycolate
TDM: Trehalose dimycolate
TMCM: Trehalose monocorynomycolate
