# Supplementary material for: Physiological roles of sigma factor SigD in Corynebacterium glutamicum
Source: BMC Microbiol. 2017 Jul 12;17:158. doi: 10.1186/s12866-017-1067-6 (PMC5508688; doi:10.1186/s12866-017-1067-6)
Supplement: Supplementary file 1 — Table S1. Bacterial strains, plasmids and oligonucleotides used in this work. Bacterial strains, plasmids and oligonucleotides used in this work are listed. (DOCX 17 kb) [file 12866_2017_1067_MOESM1_ESM.docx]

Table S1. Bacterial strains, plasmids and oligonucleotides used in this work

| Bacterial strain | Relevant characteristic | Reference or source |
| --- | --- | --- |
| E. coli |  |  |
| DH5α | F−*thi*-1 *endA*1 *hsdR*17(r−, m−) *supE*44 Δ*lacU*169 (Φ80*lacZ*ΔM15) *recA*1 *gyrA*96 *relA*1 | Bethesda Research Laboratories |
| C. glutamicum |  |  |
| WT | Wild type, ATCC 13032 | ATCC |
| ΔsigD | ΔsigD | this study |
| Plasmid | Relevant characteristic | References |
| pVWEx1 | KanR; *E. coli*-*C. glutamicum* shuttle vector for regulated gene expression (Ptac, *lacI*q, pCG1 oriVCg) | [14] |
| pVWEx1-*sigD* | KanR, pVWEx1 with *sigD* from *C. glutamicum* WT | this study |
| pK18mobsacB | KanR, *E. coli* - *C. glutamicum* shuttle vector for construction of insertion mutants in *C. glutamicum* (pK18 oriVec *sacB* *lacZ*α) | [15] |
| pK18mobsacB-Δ*sigD* | KanR, pK18mobsacB with a Δ*sigD* deletion construct | this study |
| Oligonucleotide | Sequence (5'-3') | References |
| *sigD*-fwd | GCCTGCAGGTCGACTCTAGAG***GAAAGGAGG***CCCTTCAG**TTG**GCTGATACTGAGCGCGAGCTC | [37] |
| *sigD*-rev | CGGTACCCGGGGATCTTACTTGTTCTCCTGCTGCTCAAGTGTGCTTC | [37] |
| Δ*sigD*_1_fwd | GATCTATCTAGAGATAGAACACGTCGGAGGTC | this study |
| Δ*sigD*_1_rev | CTGTGAGCATCTGCGACCTTCAAGTTCTCGCACCTTCCTG | this study |
| Δ*sigD*_2_fwd | CAGGAAGGTGCGAGAACTTGAAGGTCGCAGATGCTCACAG3 | this study |
| Δ*sigD*_2_rev | GATCTATCTAGATAACCGTGGTGTCCGAAGTG | this study |
| Δ*sigD*_genome_fwd | GATGCAGGAAGGTGCGAGAA | this study |
| Δ*sigD*_genome_rev | CTGCTCACCACCATGTAGAC | this study |
| *sigD*_qPCR_fwd | GCTGGTCAGCGATGGAAGTA | this study |
| *sigD*_qPCR_rev | TGCTGCTCAAGTGTGCTTCG | this study |
| cg0413_qPCR_fwd | CCTCGGTGGCAAGCAAATGT | this study |
| cg0413_qPCR_rev | TGTTGTGGGAAGAGTAGGGAAGTAG | this study |
| cg0420_qPCR_fwd | GATCGCTTTAGAGTTCCATCCCTTG | this study |
| cg0420_qPCR_rev | TCCTCCCTTATCTTTATTTATTCTGCCG | this study |
| cg0532_qPCR_fwd | GTGGGTTGGTTATGCGGTTCG | this study |
| cg0532_qPCR_rev | CTCGATTTCGCGCTGCTAGTG | this study |
| cg1052_qPCR_fwd | ACCGACCTTGATGAGTTGACCAG | this study |
| cg1052_qPCR_rev | TGCCCAGTCAGCGTAGAAACTAAA | this study |
| cg1181_qPCR_fwd | CATCACACCACAAACGACACGG | this study |
| cg1181_qPCR_rev | TAATGACTGCGAAATAGGCGACCAA | this study |
| cg2720_qPCR_fwd | AATCCTCGCAGCAAAGTACAGAAG | this study |
| cg2720_qPCR_rev | CACATCCTCATCACCATCATCAACA | this study |
| cg3009_qPCR_fwd | TCAAGGAAACCCTCGGCAACTA | this study |
| cg3009_qPCR_rev | GAAGTTAAGGATGGAATCGAGCAGG | this study |
| cg3178_qPCR_fwd | AGGTTATGTCTCGGAAGATGGAAGA | this study |
| cg3178_qPCR_rev | AGACCAAAGAACTCCGCATCAAAG | this study |
| cg3179_qPCR_fwd | GCTAACAACAGCCCTGAGTACATTT | this study |
| cg3179_qPCR_rev | TCGTTTGGATCATAAAGCGGCAC | this study |
| cg3180_qPCR_fwd | ACCGCGAAGATGAATGAGGAACT | this study |
| cg3180_qPCR_rev | CTGGGAGAAACCAACGATGATGAAC | this study |
| cg3182_qPCR_fwd | CTGAAATCTGGGCACTTGACGG | this study |
| cg3182_qPCR_rev | ATGGCGTTCTTATCGGCGTAGTA | this study |
| cg3186_qPCR_fwd | CTCTACGCACCAAACAACATCACC | this study |
| cg3186_qPCR_rev | ATGACAGGATCGGAGGAGGAAAGA | this study |

Sequences in bold italic represent ribosome binding sites; sequences in bold represents the translational start codons.
