# Supplementary material for: Physiological roles of sigma factor SigD in Corynebacterium glutamicum
Source: BMC Microbiol. 2017 Jul 12;17:158. doi: 10.1186/s12866-017-1067-6 (PMC5508688; doi:10.1186/s12866-017-1067-6)
Supplement: Supplementary file 2 — Table S2. RNA-seq analysis of genes differentially transcribed upon sigD overexpression or sigD disruption. The name of genes which expression levels increased under sigD overexpression (M-value > 1.0) are listed. (DOCX 18 kb) [file 12866_2017_1067_MOESM2_ESM.docx]

## Table S2. RNA-seq analysis of genes differentially transcribed upon *sigD* overexpression or *sigD* disruption

| M value for pVWEx1-*sigD*/pVWEx1 >1 | | | *sigD* over-expression | *sigD* disruption |
| --- | --- | --- | --- | --- |
| gene IDa | gene namea | Function of proteina | M-value^b^ | M-value^b^ |
| cg0079 | - | Putative secreted protein, CotH homologue | 1.2 | 0.74 |
| cg0413 | cmt1 | Trehalose corynomycolyl transferase | 1.6 | -0.41 |
| cg0420 | - | Putative glycosyltransferase | 1.0 | -0.55 |
| cg0486 | - | ABC-type transporter, ATPase and permease subunit | 2.6 | -0.53 |
| cg0532 | - | Putative glycosyltransferase | 1.9 | -0.41 |
| cg0606 | - | Putative membrane protein | 2.5 | -0.73 |
| cg0607 | - | Putative secreted protein | 3.5 | -1.8 |
| cg0696 | sigD | RNA polymerase sigma factor, ECF-family | 3.3 | -4.5 |
| cg0697 | - | Conserved protein of unknown function | 2.7 | -4.7 |
| cg1052 | cmt3 | Corynomycolyl transferase | 2.2 | -0.06 |
| cg1056 | - | Putative membrane protein | 1.2 | -0.34 |
| cg1181 | - | Glycosyltransferase, probably involved in cell wall biogenesis | 1.1 | -0.21 |
| cg1246 | - | Conserved protein of unknown function | 2.2 | -1.5 |
| cg1247 | - | Putative secreted protein | 2.3 | -1.5 |
| cg2047 | - | Putative secreted protein | 3.1 | -1.3 |
| cg2317 | - | ABC-type putative iron(III) dicitrate transporter, permease subunit | 1.6 | 0.01 |
| cg2318 | - | ABC-type putative iron(III) dicitrate transporter, substrate-binding lipoprotein | 2.9 | -1.1 |
| cg2320 | - | Putative transcriptional regulator, ArsR-family | 3.1 | -4.5 |
| cg2572 | - | Conserved protein of unknown function | 1.6 | -0.92 |
| cg2693 | - | Conserved protein of unknown function | 1.1 | 0.03 |
| cg2720 | lppS | Conserved putative secreted lipoprotein, ErfK/YbiS/YcfS/YnhG-family | 2.1 | -2.0 |
| cg2875 | - | Protein of unknown function | 1.1 | -1.1 |
| cg3138 | - | Band 7 domain-containing protein, stomatin/prohibitin homolog | 1.4 | -0.22 |
| cg3139 | - | Conserved protein of unknown function | 1.2 | 0.02 |
| cg3179 | fadD2 | Putative long-chain-fatty-acid--CoA ligase | 2.2 | -0.41 |
| cg3180 | elrF | Envelope lipids regulation factor | 1.1 | -0.10 |
| cg3181 | - | Putative secreted protein | 1.2 | -0.14 |
| cg3182 | cop1 | Trehalose corynomycolyl transferase | 1.3 | -0.04 |
| cg3186 | cmt2 | Trehalose corynomycolyl transferase | 1.0 | 0.47 |

^a^ Gene ID, gene name and function of proteins are given according to CoryneRegNet (http://coryneregnet.de/) or from references. ^b^Relative RNA levels in WT(pVWEx1-*sigD*) with 50 μM of IPTG compared to WT(pVWEx1-*sigD*) without IPTG or the Δ*sigD* strain compared to the WT strain are shown as log 2 values (M-values). IPTG was added at the beginning of the cultivation. Number of mapped reads was 7.58 million for the WT, 7.77 million for the Δ*sigD* strain, 7.93 million for WT(pVWEx1-*sigD*) without IPTG, 7.22 million for WT(pVWEx1-*sigD*) with IPTG, respectively.
